# Impact of Pharmacogenetic Markers on the Efficacy of Neoadjuvant FLOT Chemotherapy in Patients with Gastric and Gastroesophageal Junction Adenocarcinoma

**DOI:** 10.3390/ijms27094114

**Published:** 2026-05-04

**Authors:** Denis Fedorinov, Vladimir Lyadov, Marina Lyadova, Sherzod Abdullaev, Anastasia Kachanova, Anna Filatova, Dmitriy Ivashchenko, Dmitry Sychev

**Affiliations:** 1 Federal State Budgetary Research Institution «Russian Research Center of Surgery Named After Academician B.V. Petrovsky», Moscow 119991, Russia; sherzodx5@gmail.com (S.A.); dvi1991@yandex.ru (D.I.); dimasychev@mail.ru (D.S.); 2 Moscow Multidisciplinary Clinical Center “Kommunarka”, Moscow 108814, Russia; 3 Federal State Budgetary Educational Institution of Further Professional Education “Russian Medical Academy of Continuous Professional Education” of the Ministry of Healthcare of the Russian Federation, Moscow 125993, Russia; vlyadov@gmail.com (V.L.); aakachanova@yandex.ru (A.K.); 4 Moscow State Budgetary Healthcare Institution “Oncological Center No. 1 of Moscow City Hospital Named After S.S. Yudin, Moscow Healthcare Department”, Moscow 115446, Russia; dr.lyadova@gmail.com; 5 Department of Oncology, Novokuznetsk State Institute of Postgraduate Medical Education-Branch of Russian Medical Academy of Continuous Professional Education, Novokuznetsk 654005, Russia; 6 E.M. Tareev Clinic of Rheumatology, Nephrology, and Occupational Pathology. I.M. Sechenov First Moscow State Medical University (Sechenov University), Moscow 119991, Russia; filatovaanet@mail.ru; 7 Faculty of Fundamental Medicine, Lomonosov Moscow State University, Moscow 119991, Russia

**Keywords:** gene, genome, genetics, genotype, pharmacogenetics, pharmacogenomics, ERCC1, cancer

## Abstract

Despite the adoption of neoadjuvant FLOT chemotherapy as standard treatment for locally advanced gastric and gastroesophageal junction cancer, many patients fail to achieve meaningful pathological response, limiting efficacy. The aim of this study was to evaluate the impact of pharmacogenetic markers on pathological response in Russian patients receiving neoadjuvant FLOT. Thirty patients with locally advanced gastric or gastroesophageal junction adenocarcinoma received neoadjuvant FLOT followed by surgery. Polymorphisms in *CYP3A5* (rs776746), *CYP2C8* (rs10509681, rs11572080, rs1058930), *ERCC1* (rs11615), and *GSTP1* (rs1695) were analyzed. Favorable pathological response (TRG0-2) was observed in 23.3% of patients, including 3.3% complete responses. Most patients (76.7%) had minimal or no regression (TRG3-5). Clinical variables were not associated with response. *ERCC1* (rs11615) showed a significant association: patients with the Wt/Wt genotype had higher odds of achieving TRG0-2 (OR = 8.889; *p* = 0.033; 95% CI: 1.294–61.058). No associations were found for *CYP3A5, CYP2C8*, or *GSTP1*. Median PFS was 18.21 months in TRG3-5, while not reached in TRG0-2 (*p* = 0.108). No significant PFS differences by *ERCC1* genotype were observed (*p* = 0.525). *ERCC1* (rs11615) may serve as a potential pharmacogenetic marker of response to FLOT, supporting further research in personalized treatment strategies.

## 1. Introduction

There is currently no universally accepted consensus in global oncology practice regarding the optimal treatment strategy for locally advanced gastric cancer and gastroesophageal junction tumors. In East Asian countries, particularly Japan and South Korea, the standard approach traditionally consists of upfront surgical resection followed by adjuvant chemotherapy, which is largely oбycлoвлeнo established screening programs, earlier-stage diagnosis, and a high level of surgical expertise [1]. In contrast, in European countries and several Western guidelines, a perioperative strategy incorporating neoadjuvant chemotherapy is preferred, most commonly using the FLOT regimen (5-fluorouracil, leucovorin, oxaliplatin, and docetaxel), which has demonstrated a survival advantage compared with previous standard approaches [2]. These differences reflect not only regional variations in clinical practice but also the ongoing uncertainty regarding the optimal treatment strategy for this patient population.

The primary goals of neoadjuvant chemotherapy are tumor downstaging, increased resectability, and, ideally, the achievement of a complete pathological response. Pathological response is considered one of the most important prognostic factors and is closely associated with long-term clinical outcomes. In the FLOT4-AIO trial, the FLOT regimen increased the rate of major pathological response (defined as ≤10% residual tumor cells) to 37% compared with 23% in the ECF/ECX group, which was accompanied by an improvement in median overall survival from 35 to 50 months and an increase in 5-year overall survival from 36% to 45% [2]. Subsequent studies have confirmed that patients achieving complete or major tumor regression, as assessed by the Becker or Mandard grading systems, demonstrate significantly improved progression-free and overall survival compared with those exhibiting minimal or no response [3]. Thus, the degree of pathological response may be regarded as a surrogate marker of treatment efficacy and disease prognosis.

However, despite the standardization of treatment approaches and the introduction of modern chemotherapy regimens, it remains unclear why only a subset of patients achieves a meaningful pathological response. Several studies have attempted to identify clinicopathological and molecular factors associated with the efficacy of neoadjuvant therapy. Tumor histology, degree of differentiation, anatomical location, molecular subtypes (including microsatellite instability status), as well as the expression of specific proteins and genetic alterations, have all been shown to influence the probability of response [4]. In particular, tumors characterized by microsatellite instability have been shown to exhibit reduced sensitivity to cytotoxic therapy and may derive limited benefit from neoadjuvant treatment. However, these factors do not allow for sufficiently accurate prediction of individual treatment response, thereby limiting the potential for effective personalization of therapy.

In recent years, increasing attention has been focused on pharmacogenetic markers that may influence both the efficacy and tolerability of chemotherapy. Genetic polymorphisms affecting drug metabolism, transport, and DNA repair pathways are known to contribute to interindividual variability in treatment response. For the components of the FLOT regimen, several potentially relevant pharmacogenetic associations have been described, including DPYD variants affecting fluoropyrimidine metabolism [5], *ERCC1* polymorphisms associated with the efficacy of platinum-based agents [6], and variants in *CYP3A* genes and *ABC* transporters involved in the metabolism and elimination of taxanes [7]. At present, pharmacogenetic markers have not been incorporated into routine decision-making algorithms for neoadjuvant therapy in this setting, underscoring the relevance of further research in this area.

The aim of the present study was to evaluate the impact of pharmacogenetic markers CYP3A5*3 (rs776746), *CYP2C8* (rs10509681), CYP2C8*3 (rs11572080), CYP2C8*4 (rs1058930), *ERCC1* (rs11615), and *GSTP1* (rs1695) on the probability of achieving a pathological response in patients with gastric and gastroesophageal junction adenocarcinoma receiving neoadjuvant FLOT chemotherapy in a Russian patient population.

The selection of pharmacogenetic markers in the present study was based on their established or potentially significant roles in the pharmacokinetics and pharmacodynamics of the individual components of the FLOT regimen. ERCC1 is a key component of the nucleotide excision repair system and plays a central role in the removal of platinum-induced DNA damage, thereby influencing tumor sensitivity to oxaliplatin. CYP3A5 is one of the principal enzymes responsible for the metabolism of docetaxel, while CYP2C8 contributes to taxane biotransformation. GSTP1 is involved in cellular detoxification processes, including conjugation reactions that may affect the efficacy of platinum-based chemotherapy. Taken together, these genes represent biologically plausible candidates for predicting variability in treatment response to the FLOT regimen.

Despite the widespread adoption of the FLOT regimen as a standard of care, there remains a lack of validated pharmacogenetic markers capable of predicting pathological response in the neoadjuvant setting. Existing studies are limited in size, heterogeneous in design, and often focus on single agents rather than combination regimens. Therefore, the identification of clinically relevant pharmacogenetic predictors of response to FLOT represents an important unmet need in the personalization of treatment for gastric and gastroesophageal junction cancer.

## 2. Results

Within the present study, all included patients underwent pharmacogenetic testing for the following polymorphic variants: CYP3A5*3 (rs776746), *CYP2C8* (rs10509681), CYP2C8*3 (rs11572080), CYP2C8*4 (rs1058930), *ERCC1* (rs11615), and *GSTP1* (rs1695). Analysis of genotype distributions demonstrated that all studied markers were in Hardy–Weinberg equilibrium, indicating the absence of systematic bias in cohort selection, the reliability of genotyping procedures, and the representativeness of the study population with respect to the investigated genetic variants. These findings support the validity of the data and their suitability for subsequent analyses of associations between genotypes and clinical outcomes. The results are presented in Table 1.

Assessment of treatment response using the pathological tumor regression grading system revealed marked heterogeneity in outcomes. A complete pathological response (TRG0) was achieved in 1 patient (3.3%). A major response (TRG1) was observed in 3 patients (10.0%), while an equal proportion (n = 3; 10.0%) demonstrated moderate tumor regression (TRG2). Limited pathological response (TRG3) was identified in 6 patients (20.0%).

**Figure 1:**
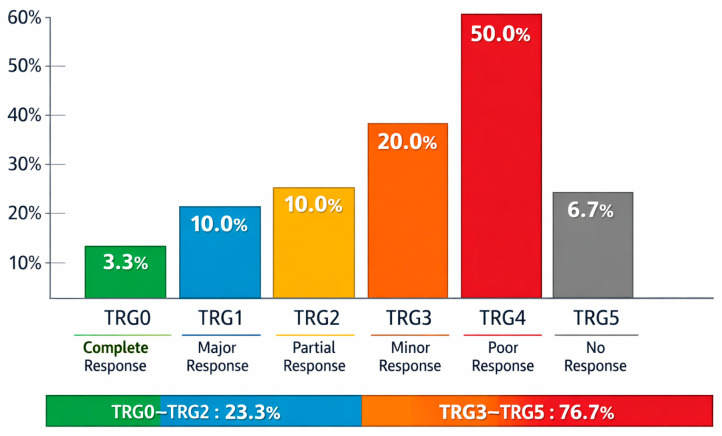
Pathological Tumor Regression Grade (TRG) Distribution Following Neoadjuvant Chemotherapy.

At the same time, the majority of patients exhibited little or no response to therapy: TRG4 was recorded in 15 patients (50.0%), and TRG5 in 2 patients (6.7%). Overall, the rate of objective pathological response (TRG0–TRG2) was 23.3%, whereas 76.7% of patients showed minimal or no response to neoadjuvant chemotherapy. These findings indicate substantial interindividual variability in tumor sensitivity to treatment. The data are illustrated in Figure 1.

An analysis was performed to assess the impact of clinicodemographic and tumor-related characteristics on the degree of pathological response to neoadjuvant chemotherapy. None of the evaluated factors demonstrated a statistically significant association with the likelihood of achieving a favorable pathological response.

In contrast, a genetic factor, specifically the *ERCC1* (rs11615) polymorphism, was found to have a significant effect on tumor regression. According to the data, statistically significant differences in TRG distribution were observed across *ERCC1* genotypes (*p* = 0.033). Patients with the Wt/Wt genotype had 8.889-fold higher odds of achieving a favorable pathological response (TRG0–2) compared with those carrying Wt/mut or mut/mut genotypes; this difference was statistically significant (95% CI: 1.294–61.058). These findings suggest a potential prognostic role of *ERCC1* polymorphism in predicting response to neoadjuvant FLOT chemotherapy.

**Figure 2:**
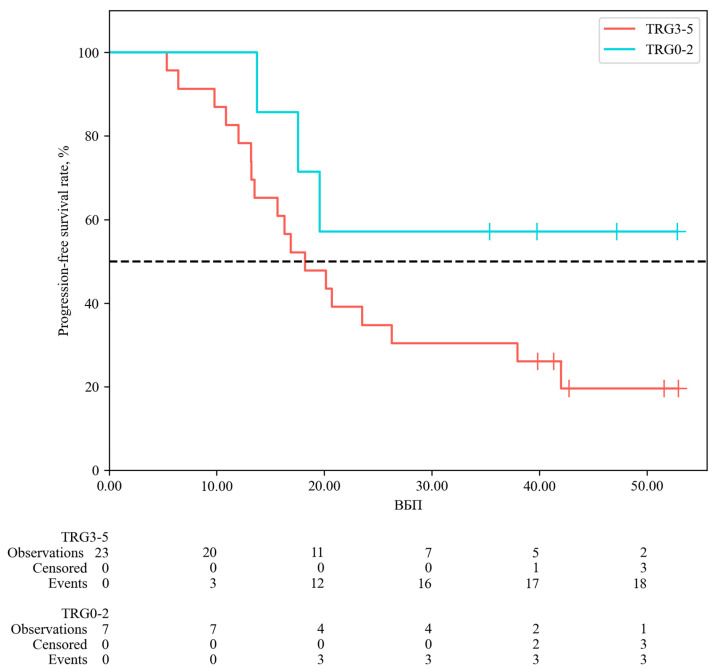
Progression-free survival curve conditioning on TRG.

Survival analysis according to the degree of pathological response showed that the median progression-free survival in patients with TRG3–5 was 18.21 months from the start of follow-up (95% CI: 13.22–26.27), whereas the median was not reached in the TRG0–2 group. Although a trend toward a more favorable prognosis was observed in patients with a pronounced pathological response, the differences between groups, assessed using the likelihood ratio test, did not reach statistical significance (*p* = 0.108). The data are illustrated in Figure 2.

Analysis of progression-free survival according to *ERCC1* (rs11615) genotype showed that the median survival was not reached in patients with the Wt/Wt genotype, whereas it was 20.15 months (95% CI: 12.03–26.27) in the Wt/mut group and 18.21 months (95% CI: 13.22–42.02) in the mut/mut group. However, differences in progression-free survival between genotype groups, assessed using the likelihood ratio test, were not statistically significant (*p* = 0.525).

## 3. Discussion

The findings of the present study highlight the ongoing clinical challenge of heterogeneous response to neoadjuvant chemotherapy in patients with gastric and gastroesophageal junction adenocarcinoma. Despite the use of the contemporary standard FLOT regimen, a favorable pathological response (TRG0–2) was achieved in only 23.3% of patients, while the majority (76.7%) exhibited minimal or no response to treatment. These results are consistent with data from large randomized trials, including FLOT4-AIO, where the rate of major tumor regression did not exceed 35–40%, underscoring the need for predictive markers of treatment efficacy.

In the present study, we evaluated the impact of pharmacogenetic markers involved in the metabolism and mechanisms of action of FLOT components, including *CYP3A5*, *CYP2C8, ERCC1*, and *GSTP1*. These genes represent key elements of the pharmacokinetics and pharmacodynamics of taxanes, platinum agents, and fluoropyrimidines, making them plausible candidates for predicting treatment response.

The *CYP3A5* gene (rs776746, *3 polymorphism) encodes a cytochrome P450 isoenzyme involved in the metabolism of taxanes, including docetaxel. The *3 allele is associated with reduced or absent enzymatic activity, which may lead to increased drug exposure and, theoretically, enhanced antitumor efficacy or toxicity. However, the available evidence remains inconsistent: some studies have shown that carriage of nonfunctional *CYP3A5* variants does not significantly affect clinical outcomes of taxane-based therapy [8], whereas others suggest an association with drug pharmacokinetics without a clear impact on treatment efficacy [9]. In our study, no statistically significant association was observed between this polymorphism and the degree of pathological response, which is consistent with the majority of published data.

The *CYP2C8* gene (rs10509681, rs11572080, rs1058930) is also involved in the metabolism of taxanes, primarily paclitaxel, although its role in docetaxel metabolism appears to be less pronounced. The CYP2C8*3 and CYP2C8*4 polymorphisms are associated with altered enzymatic activity and may influence the pharmacokinetics of these agents [10]. However, clinical studies have not demonstrated a consistent association between *CYP2C8* variants and treatment efficacy in solid tumors [11]. Our findings, which did not demonstrate a significant association between these polymorphisms and pathological response, support the limited clinical relevance of these markers in the context of the FLOT regimen.

The *GSTP1* gene (rs1695) encodes glutathione S-transferase, an enzyme involved in the detoxification of cytotoxic agents, including platinum compounds. The rs1695 polymorphism is associated with altered enzymatic activity and may influence tumor sensitivity to chemotherapy. Several studies have reported that variant *GSTP1* alleles are associated with improved response to platinum-based therapy [12]; however, the overall evidence remains inconsistent. In our study, no statistically significant association between GSTP1 and pathological response was identified, which may be attributable to the limited sample size or the multifactorial nature of drug effects within the FLOT regimen.

Particular attention should be given to the *ERCC1* gene (rs11615), which encodes a protein involved in the nucleotide excision repair pathway and plays a key role in the removal of DNA damage induced by platinum-based agents. Reduced expression or functional activity of *ERCC1* has been associated with increased sensitivity of tumor cells to oxaliplatin. Several studies have suggested that *ERCC1* polymorphisms may be associated with the efficacy of platinum-based chemotherapy in gastric cancer and other solid tumors [13]. In particular, the rs11615 variant has been associated with differences in treatment response and survival outcomes. In our study, for the first time in a cohort of patients receiving neoadjuvant FLOT chemotherapy, a statistically significant association was demonstrated between *ERCC1* genotype and the likelihood of achieving a favorable pathological response: patients with the Wt/Wt genotype had 8.889-fold higher odds of achieving TRG0–2 compared with carriers of mutant alleles (*p* = 0.033). This finding is of clinical relevance and is consistent with the established role of *ERCC1* in mechanisms of resistance to platinum-based agents.

Analysis of progression-free survival showed an expected trend toward improved outcomes in patients with a favorable pathological response (TRG0–2); however, the differences did not reach statistical significance (*p* = 0.108). Similarly, although patients with the *ERCC1* Wt/Wt genotype demonstrated more favorable survival outcomes, no statistically significant differences in progression-free survival were observed between genotype groups (*p* = 0.525). Although a statistically significant association was observed between *ERCC1* rs11615 and pathological response, no corresponding differences in progression-free survival were identified. This may reflect the limited statistical power of the study, as survival analyses generally require larger sample sizes and a sufficient number of events to detect meaningful differences.

The integration of pharmacogenetic markers into clinical decision-making represents a promising step toward the personalization of neoadjuvant chemotherapy in gastric and gastroesophageal junction cancer. Identification of genetic variants associated with treatment response may allow for improved patient stratification and optimization of therapeutic strategies, particularly in the context of multi-agent regimens such as FLOT. In this setting, pharmacogenetic profiling could contribute to the selection of patients most likely to benefit from platinum-based chemotherapy, while also supporting the development of alternative approaches for those with a lower expected response.

This study has several limitations that should be considered when interpreting the results. First, the relatively small sample size may have limited the statistical power of the analysis and contributed to the lack of significant differences in certain outcomes, including progression-free survival. In addition, the analysis was restricted to a selected panel of pharmacogenetic markers and does not capture the full complexity of genetic determinants of treatment response. Variability in the assessment of pathological response may also have influenced the findings. The identified association between the *ERCC1* rs11615 polymorphism and pathological response represents a clinically relevant finding that is supported by the applied statistical analysis and consistent with the biological role of ERCC1 in DNA repair mechanisms. The wide confidence interval observed for the *ERCC1* rs11615 polymorphism indicates a degree of uncertainty in the effect estimate and highlights the need for cautious interpretation. The limited sample size may have reduced the statistical power to detect associations for other polymorphisms included in the analysis. While multivariate modeling may provide additional insights in larger datasets, its application in the present study is constrained by the risk of overfitting due to the small number of observations relative to the number of potential covariates. At the same time, this observation should be further validated in larger prospective cohorts to confirm its robustness and generalizability. Future studies may benefit from the integration of advanced analytical approaches aimed at elucidating the biological mechanisms underlying treatment response. In particular, Mendelian randomization (MR) represents a promising methodological framework in which genetic variants can be used as instrumental variables to explore potential causal relationships between molecular biomarkers and clinical outcomes. The application of MR in pharmacogenetic oncology research may provide additional insights into whether *ERCC1* polymorphisms directly influence sensitivity to platinum-based chemotherapy or reflect broader biological processes [14].

## 4. Materials and Methods

From January 2021 to February 2024, perioperative treatment for gastric cancer was administered to 30 patients in the Chemotherapy Department No. 1 of Oncology Center No. 1 at S.S. Yudin City Clinical Hospital, Moscow. All patients were informed about the procedures and potential risks and provided written informed consent for study participation and for the processing of personal data. Pharmacogenetic testing was performed in all patients prior to the first course of chemotherapy. The main clinicodemographic characteristics of the study cohort are presented in Table 2.

Treatment was administered in accordance with current national clinical guidelines for gastric cancer. All patients initially underwent diagnostic laparoscopy to exclude distant metastases, after which four cycles of perioperative chemotherapy were planned every 14 days using the FLOT regimen (docetaxel 50 mg/m^2^, oxaliplatin 85 mg/m^2^, calcium folinate 200 mg/m^2^, and 5-fluorouracil 2600 mg/m^2^ administered as a 24 h infusion).

Allelic variants of genes were determined by polymerase chain reaction at the Russian Medical Academy of Continuous Professional Education.

A volume of 4–6 mL of venous blood was obtained from the antecubital vein and collected into VACUETTE^®^ tubes (Greiner Bio-One, Kremsmünster, Austria) containing EDTA-K2 or EDTA-K3 for subsequent genomic DNA extraction. The collected samples were stored at −80 °C prior to processing. Genomic DNA was extracted from whole blood using the S-Sorb kit based on silica sorbent technology (Sintol LLC, Moscow, Russia). DNA concentration was determined using a NanoDrop 2000 microvolume spectrophotometer (Thermo Fisher Scientific, Veterans Memorial Hwy, Bohemia, NY, USA).

Genotyping of polymorphic variants was performed using commercial TaqMan^®^ SNP Genotyping Assays in combination with TaqMan Universal Master Mix II No UNG (Applied Biosystems, Veterans Memorial Hwy, Bohemia, NY, USA). PCR amplification was carried out in a total reaction volume of 20 μL. In accordance with the manufacturer’s protocol, 1 μL of TaqMan^®^ SNP Genotyping Assay (pre-diluted 1:40) was combined with 10 μL of TaqMan Universal Master Mix II No UNG and 9 μL of RNase-free water. Subsequently, 5 μL of DNA from each sample was added to the reaction mixture. Real-time PCR for SNP genotyping was conducted using the CFX96 Touch Real-Time PCR Detection System with CFX Manager software version 3.0 (Bio-Rad, Hercules, CA, USA).

Statistical analysis was performed using StatTech v. 4.12.5 (Developer-StatTech LLC, Moscow, Russia).

Quantitative variables were assessed for normality using the Shapiro–Wilk test. Variables with a normal distribution were summarized as means (M) and standard deviations (SD), with 95% confidence intervals (95% CI) reported to reflect the precision of the estimates. Categorical variables were presented as absolute counts and percentages. Comparisons between two groups for normally distributed continuous variables with equal variances were performed using Student’s *t*-test. Proportions in contingency tables were compared using Fisher’s exact test when the minimum expected cell count was less than 10. Odds ratios (OR) with 95% confidence intervals were calculated as a measure of effect size for categorical comparisons. In cases with zero cell counts, the Haldane–Anscombe correction was applied for the calculation of odds ratios. The estimation of the patient survival function was conducted using the Kaplan–Meier method. Survival analysis of patients was conducted using Cox regression, which involves predicting the instantaneous risk of an event occurring at a specific point in time (hazard). Hazard ratios (HR) with 95% confidence intervals (95% CI) were calculated, and the statistical significance of the association with predictors was evaluated. Differences were considered statistically significant at *p* < 0.05.

## 5. Conclusions

Thus, the most notable finding of the present study is the identification of a statistically significant association between the *ERCC1* (rs11615) polymorphism and the probability of achieving a favorable pathological response to neoadjuvant FLOT chemotherapy. In contrast to the other genes analyzed, which did not demonstrate clinically meaningful effects, *ERCC1* may represent a promising pharmacogenetic marker with potential utility for patient stratification and treatment personalization. These findings require validation in larger prospective cohorts and may serve as a basis for the development of individualized therapeutic strategies in patients with gastric cancer.

## Figures and Tables

**Table 1:** Distribution of Genotypes in the Study Cohort.

Parameter	Genotypes	N, %	X^2^	*p*-Value
CYP3A5*3 rs776746	Wt\Wt	26 (86.7%)	0.15	0.695
	Wt\mut	4 (13.3%)		
*CYP2C8* rs10509681	Wt\Wt	26 (86.7%)	0.15	0.695
	Wt\mut	4 (13.3%)		
CYP2C8*3 rs11572080	Wt\Wt	26 (86.7%)	0.15	0.695
	Wt\mut	4 (13.3%)		
CY2C8*4 rs1058930	Wt\Wt	29 (96.7%)	0.008	0.926
	Wt\mut	1 (3.3%)		
*ERCC1* rs11615	Wt\Wt	7 (23.3%)	1.265	0.260
	Wt\mut	18 (60.0%)		
	Mut\Mut	5 (16.7%)		
*GSTP1* rs1695	Wt\Wt	15 (50.0%)	0.068	0.794
	Wt\mut	12 (40.0%)		
	Mut\Mut	3 (10.0%)		

**Table 2:** Clinicodemographic characteristics of the study cohort.

Parameter	Value
Sex (male/female), n (%)	23 (77%)/7 (23%)
Mean age, years ± SD (min–max)	61.73 ± 9.50 (43–76)
T2	7 (23.4%)
T3	15 (50%)
T4a	8 (26.6%)
N0	11 (36.7%)
N1	12 (40%)
N2	7 (23.3%)
Well/moderately differentiated	6 (20%)
Poorly differentiated	20 (66.7%)
Unknown	4 (13.3%)
ECOG performance status (0/1), n (%)	15 (50%)\15 (50%)

## Data Availability

The datasets generated and analyzed during this study are not publicly available due to ethical restrictions and patient confidentiality protections under Russian Federation laws on personal data protection (Federal Law No. 152-FZ). However, anonymized data supporting the findings may be made available upon reasonable request from qualified researchers, subject to approval by the Local Ethics Committee of the Russian Medical Academy of Continuous Professional Education (contact: rmapo@rmapo.ru). Requests should include a detailed research proposal and data protection plan.

## Abbreviations

The following abbreviations are used in this manuscript:

ABC	ATP-binding cassette transporters
CI	Confidence Interval
CYP	Cytochrome P450
DPYD	Dihydropyrimidine dehydrogenase
ECF	Epirubicin, Cisplatin, Fluorouracil
ECX	Epirubicin, Cisplatin, Capecitabine
ERCC1	Excision Repair Cross-Complementation Group 1
FLOT	Fluorouracil, Leucovorin, Oxaliplatin, Docetaxel
GSTP1	Glutathione S-transferase
MR	Mendelian randomization
OR	Odds Ratio
SD	Standard Deviation
TRG	Tumor Regression Grade
Wt	Wild type
mut	Mutant allele
